# To Subscribers

**Published:** 1871-04

**Authors:** 


### TO SUBSCRIBERS.

As will be seen, the subscription price of the Journal is $3. This is barely sufficient to cover the expense of publication, and every copy received and read during the year, without the subscription price being forwarded, takes three dollars from our pocket, besides the labor and responsibility of constant editorial duties. To individuals who owe but three dollars there seems to be no great interest at stake, and the failure to respond, does not appear likely to effect any one seriou-ly. It must be remembered, however, that it requires the aggregate of all these debts to make the sum actually expended in money, to afford this year's reading, and unless it be sent on, the poor editors and proprietors must suffer; for the publisher requires, and will have his pay monthly.

This number of the Journal will be sent to some who have not signified their willingne-s to subscribe, and it is expected of all such who do not wish it at the very low price we prepose, to return the copy sent them, otherwise their names will stand in the list of regular subscribers.

---

We regret that the Electrotype for Dr. Love's Nasal Spritz, intended to accompany his article, could not be obtained in time to be presented therewith. It will appear in our next issue with a paper on "*Special Diseases of the Nasal Cavities.*"

